# Supplementary material for: A bispecific CD40 agonistic antibody allowing for antibody-peptide conjugate formation to enable cancer-specific peptide delivery, resulting in improved T Cell proliferation and anti-tumor immunity in mice
Source: Nat Commun. 2024 Nov 5;15:9542. doi: 10.1038/s41467-024-53839-5 (PMC11538452; doi:10.1038/s41467-024-53839-5)
Supplement: Supplementary file 1 — Supplementary Information [file 41467_2024_53839_MOESM1_ESM.pdf]

A bispecific CD40 agonistic antibody allowing for antibody-peptide conjugate formation to enable cancer-specific peptide delivery, resulting in improved T proliferation and anti-tumor immunity in mice

Co-First authors:

Aman Mebrahtu

Email: Amanme@kth.se

KTH Royal Institute of Technology, Department of Protein Science, School of Engineering Sciences in Chemistry, Biotechnology and Health, Stockholm, Sweden

Ida Laurén

Email: ida.lauren@farmaci.uu.se

Department of Pharmacy, Science for Life Laboratory, Uppsala University, Uppsala, Sweden

Corresponding authors:

Prof. Sara Mangsbo

Email: sara.mangsbo@farmaci.uu.se

Department of Pharmacy, Science for Life Laboratory, Uppsala University, Uppsala, Sweden

Prof. Johan Rockberg

Email: johanr@biotech.kth.se

KTH Royal Institute of Technology, Department of Protein Science, School of Engineering Sciences in Chemistry, Biotechnology and Health, Stockholm, Sweden

Supplementary Figures

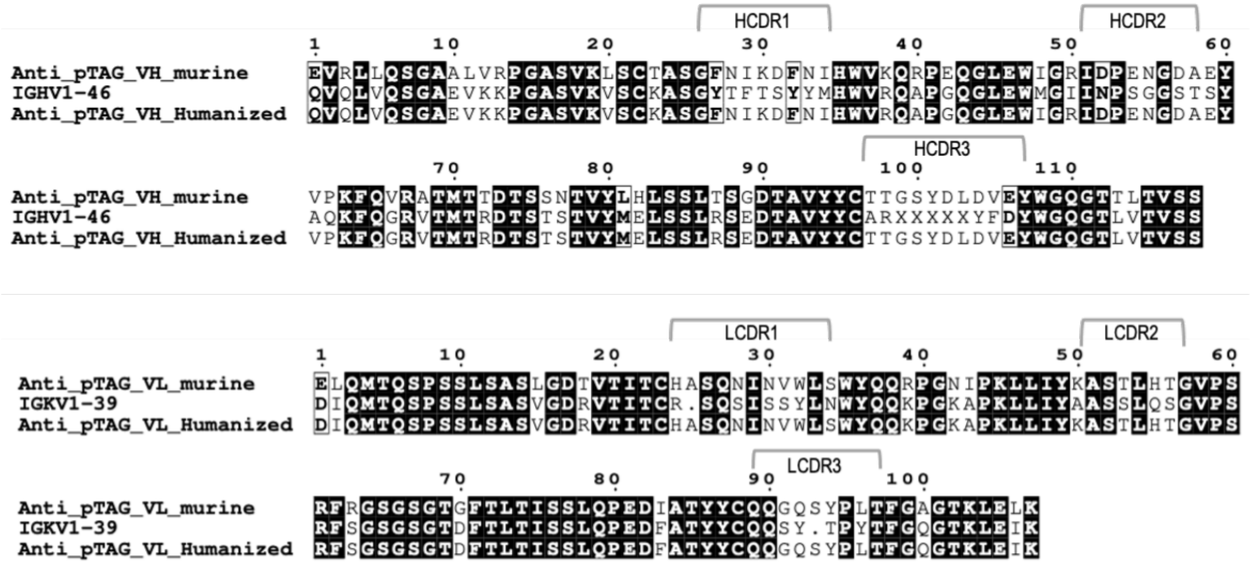

Supplementary Figure 1. Sequence alignment of humanized anti-pTag scFv. The WT murine framework illustrated with the CDRs on respective variable region and sequence aligned with suggested grafting donor framework and the resulting framework sequence with grafted CDRs.

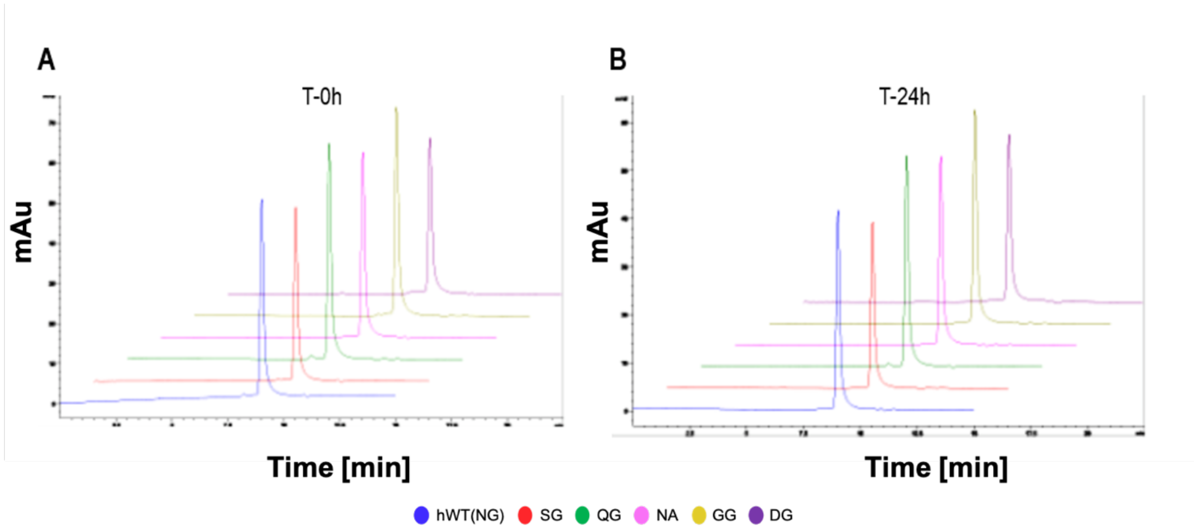

Supplementary Figure 2. SEC profile of humanized anti-pTag scFv and deamidation site removed mutants. A) SEC profile at time point 0h. **B)** SEC profile post incubation at 37 °C for 24h.

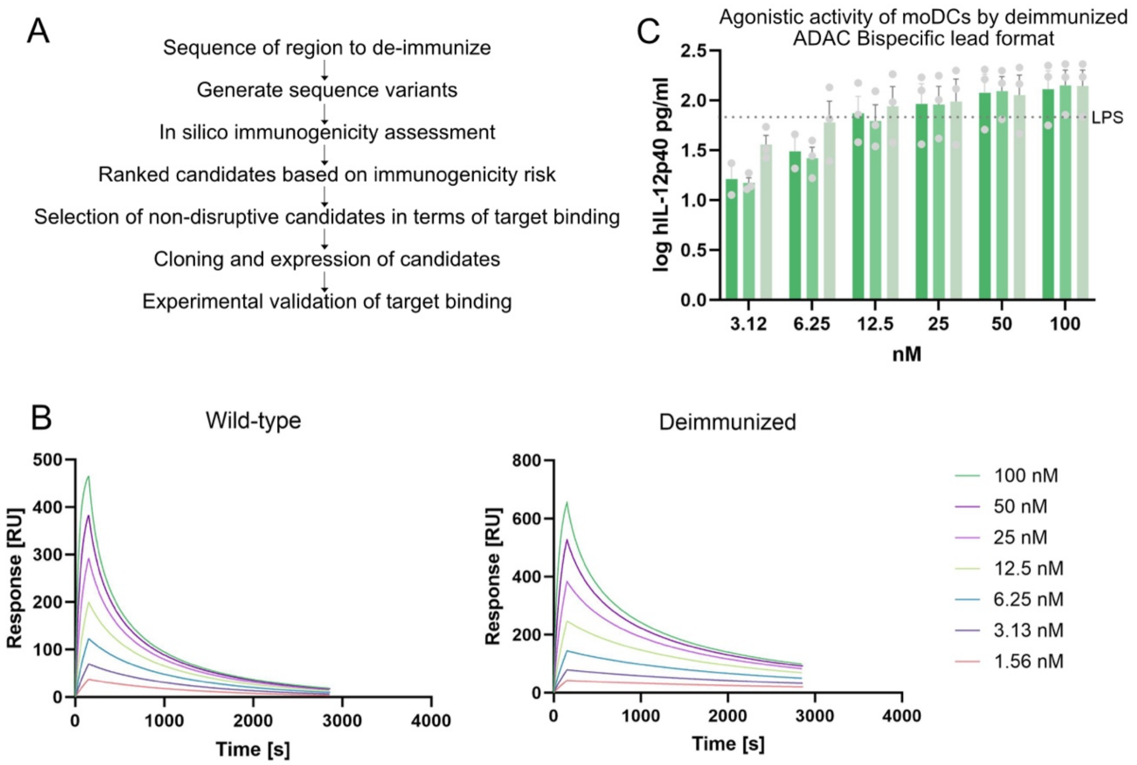

Supplementary Figure 3. Deimmunization of novel anti-CD40 mAb clone A9. A) Schematic workflow of deimmunization strategy. **B)** SPR analysis of binding to CD40. WT=Wildtype, DI=Deimmunized. **C)** Agonistic activity of deimmunized clones was performed by incubation with immature moDCs for 48h and evaluated for secreted IL-12p40 levels in the supernatant quantified by ELISA, n=3 (independent donors) Data shown as mean with ± SEM.

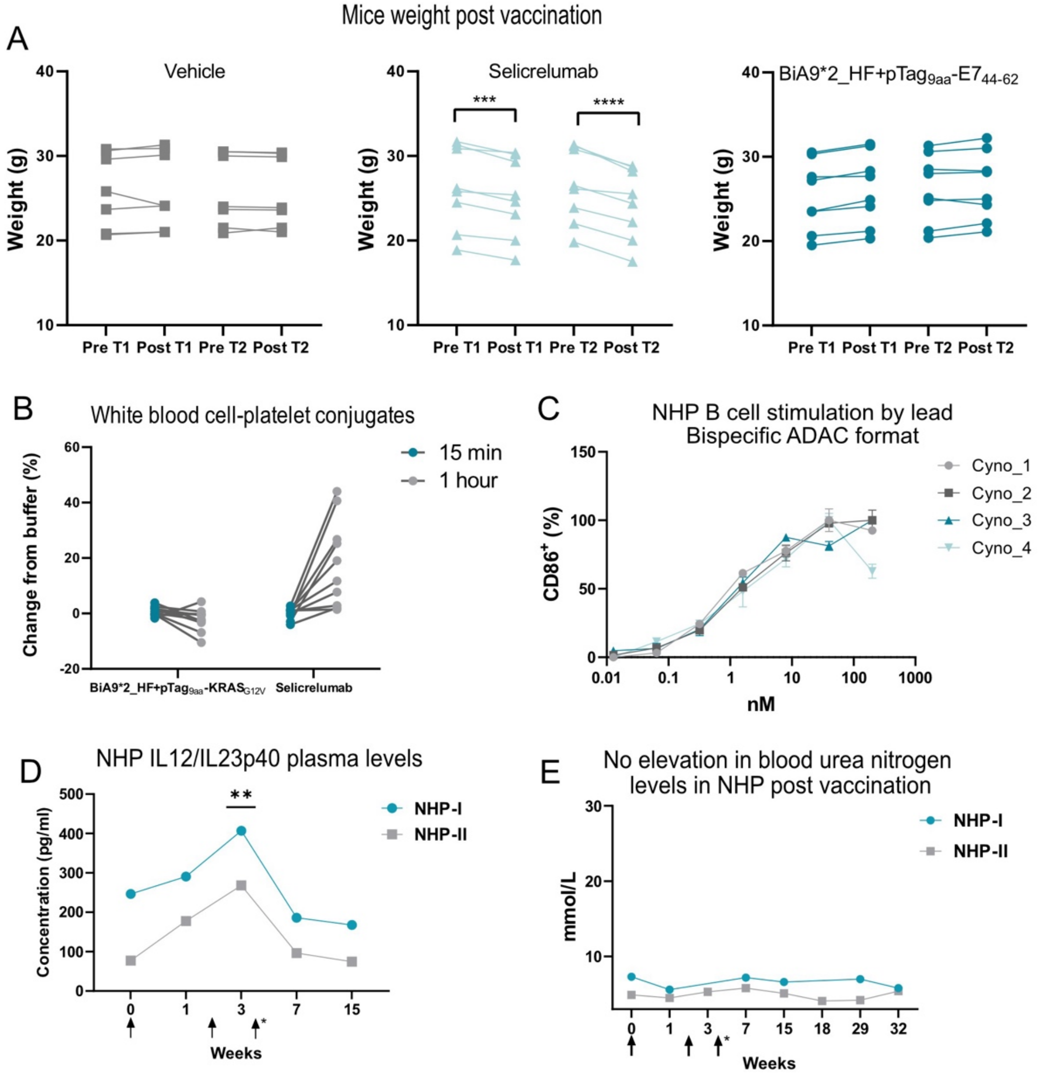

Supplementary Figure 4. Toxicity parameters in several model species. A) Mouse weight pre and post first and second treatment dose. Vehicle n=7, Selicrelumab n= 8 and BiA9*2_HF+ pTag$_{9aa}$-E7$_{44-62}$ n=8, in Biocytogen mice. ***p=0.007 and ****p=<0.0001 calculated with two-tailed paired t-test. **B)** White blood cell-platelet conjugate frequency in ID.Flow illustrating the change of white blood cell-platelet conjugates as compared to buffer alone at 15 min and 1 hour. n=10 (independent donors). **C)** Stimulated PBMCs with BiA9*2_HF from cynomolgus donors for 24h and measured CD86 expression on CD20$^+$ B cells illustrated as percentage increase of CD86 MFI expression compared to medium control. n=4 (independent individuals). **D and E)** Quantified levels of IL-12/IL-23p40 and blood urea nitrogen in plasma of immunized NHPs at indicated time-points. Arrows indicate time of vaccination and * means that only NHP-II got the third treatment. **p=0.0081 calculated with two-way ANOVA with Tukey's multiple comparison test. Data shown as mean with ± SEM

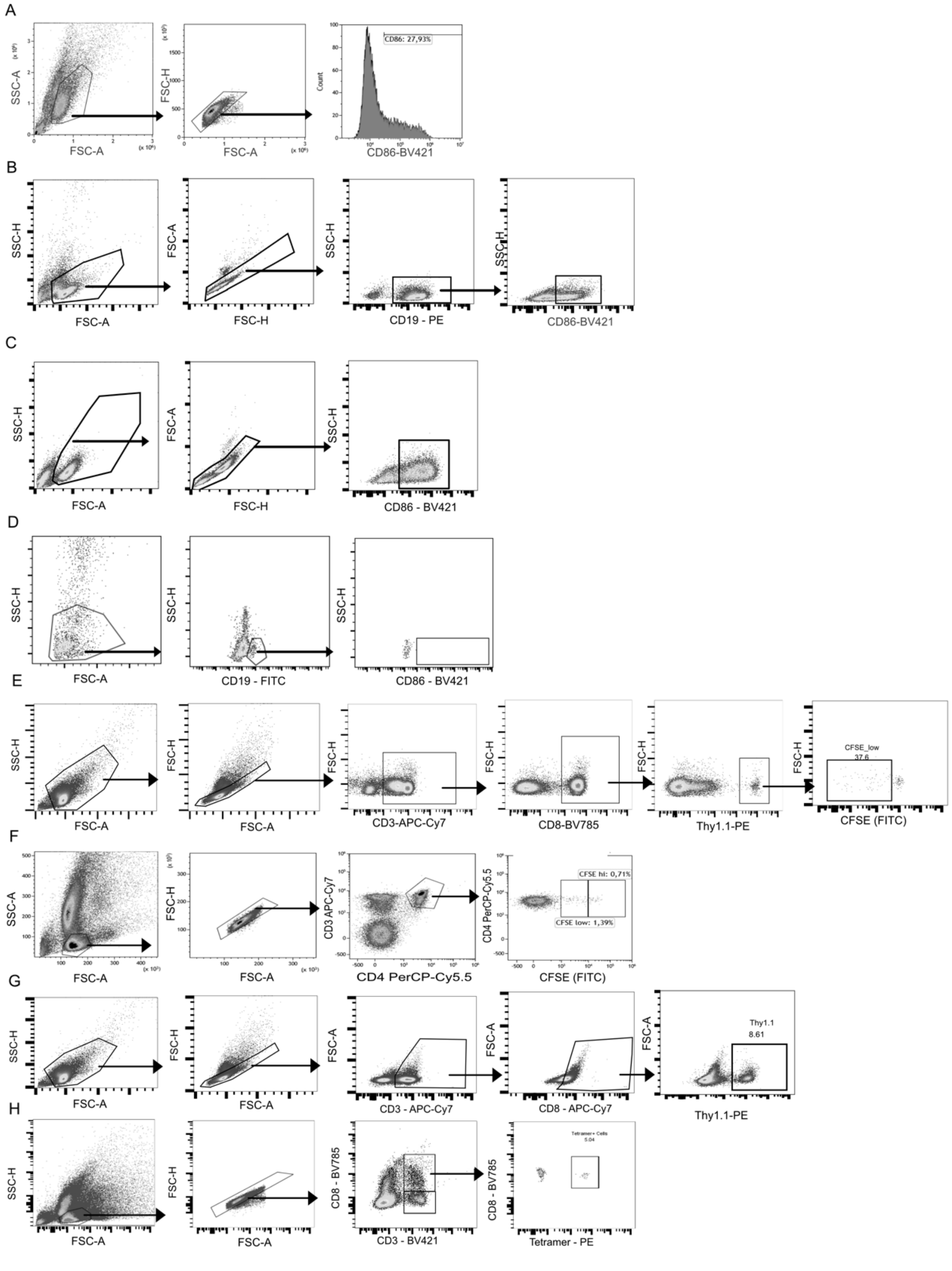

Supplementary Figure 5. Gating strategies for flow cytometry analysis. A) Gating strategy for CD86 expression on moDC presented in figure 2C. B) Gating strategy for CD86 expression on human B cells presented in figure 2D. C) Gating strategy for CD86 expression on moDC presented in figure 5B. D) Gating

strategy for CD86 expression on human B cells presented in figure 5D. E) Gating strategy to identify antigen specific CD8 T cell proliferation, defined as CFSElow, of transferred PMEL-1 lymphocytes with the Thy1.1 congenic marker presented in figure 6A. F) Gating strategy to identify antigen specific CD4 T cell proliferation, defined as CFSElow, of transferred OT-II lymphocytes presented in figure 6B. G) Gating strategy to identify antigen specific CD8 T cell expansion of transferred PMEL-1 lymphocytes with the Thy1.1 congenic marker presented in figure 6C. H) Gating strategy to identify endogenous antigen specific CD8 T cells by tetramer staining presented in figure 7C.

Supplementary Tables

Supplementary Table 1. FACS antibodies summary. Table overview of FACS antibodies used within the scope of the work with marker, fluorophore, Antibody clone, final dilutions used, catalogue numbers and vendors stated.

FACS Antibody Markers, Conjugated Fluorophore, clones and dilutions summary					
**Mouse**					
Marker	Fluorophore	Clone	Dilution	Catalogue number	Vendor
CD90.1 (Thy1.1)	PE	OX-7	1:800	202524	Biolegend
CD3	BV421	17A2	1:50	100228	Biolegend
	APC-Cy7	17A2	1:100	100222	Biolegend
CD8	APC	53-6.7	1:50	100712	Biolegend
	Pacific blue	53-6.7	1:400	100725	Biolegend
	BV785	53-6.7	1:400	100750	Biolegend
CD4	PerCP-Cy5.5	RM4-4	1:400	116012	Biolegend
CD11b	PerCP	M1/70	1:200	101230	Biolegend
CD11c	PE	N418	1:200	117308	Biolegend
I-A/I-E	APC	M5/114.15.2	1:400	107614	Biolegend
CD86	BV421	GL-1	1:200	105008	Biolegend
H-2D(b)	PE	HPV16 E7 49-57	1:25	NIH Tetramer Core Facility	
**Human**					
HLA-DR	PE-Cy5	L243	1:100	307608	Biolegend
CD83	APC	HB15e	1:100	305312	Biolegend
CD86	BV421	IT2.2	1:100	305426	Biolegend
	PE	BU63	1:200	ab77226	Abcam
CD14	APC-Cy7	63D3	1:100	367108	Biolegend
CD1a	PE	HI149	1:100	300106	Biolegend
CD19	APC-Cy7	HIB19	1:200	302218	Biolegend
	FITC	BU63	1:200	ab1167	Abcam
CD20	FITC	2H7	1:200	980202	Biolegend
HLA-A3	PE	GAP.A3	1:200	566605	BD Biosciences

Supplementary Table 2. Sequence summary pTag peptides. Sequence list overview of pTag peptides used herein. Underscored sequences indicate pTag sequence. Sequences in bold or italic represent $CD8^+$ and $CD4^+$ epitopes, respectively. Sequences in both bold and italic indicate overlapping parts of $CD8^+$ and $CD4^+$ epitopes.

pTag peptides amino acid sequences

Peptide name	Sequence
Original 18aa peptide - pTag$_{18aa}$	FIGITELKKLESKINKVF
pTag$_{16aa}$	FIGITELKKLESKINK
pTag$_{14aa}$	FIGITELKKLESKI
pTag$_{12aa}$	FIGITELKKLES
pTag$_{10aa}$	FIGITELKKL
pTag$_{9aa}$	FIGITELKK
pTag$_{8aa}$	FIGITELK
pTag$_{9aa}$ - pp65$_{489-510}$	FIGITELKKAGILAR**NLVPMVATV**QGQNLKY
pTag$_{9aa}$ - OVA$_{323-339}$	FIGITELKK*ISQAVHAAHAEINEAGR*
pTag$_{11aa}$ - gp100$_{20-39}$	FIGITELKKLEAVGAL**KVPRNQDWL**GVPRQL
pTag$_{10aa}$ - gp100$_{20-39}$	FIGITELKKLAVGAL**KVPRNQDWL**GVPRQL
pTag$_{9aa}$ - gp100$_{20-39}$	FIGITELKKAVGAL**KVPRNQDWL**GVPRQL
pTag$_{9aa}$ - E7$_{44-62}$	FIGITELKKQAEP*D**RAHYNIVTF**CCKCD*
pp65$_{489-510}$	AGILAR**NLVPMVATV**QGQNLKY
gp100$_{20-39}$	AVGAL**KVPRNQDWL**GVPRQL
OVA$_{323-339}$	*ISQAVHAAHAEINEAGR*
E7$_{44-62}$	QAEP*D****RAHYNIV*****TF***CCKCD*
pTag$_{9aa}$ - OVA$_{252-264}$	FIGITELKKAAYLEQLE**SIINFEKL**AAAAAK
pTag$_{9aa}$-KRAS$_{G12V}$	FIGITELKKYKL**VVVGAVGVGK**SALT
pTag$_{9aa}$-KRAS$_{G12D}$	FIGITELKKYKL**VVVGADGVGK**SALT
pTag$_{9aa}$ - Adpgk$_{295-313}$ (R304M)	FIGITELKKHLEL**ASMTNMELM**SSIVHQ
pTag$_{7aa}$ - Adpgk$_{295-313}$ (R304M)	GITELKKHLEL**ASMTNMELM**SSIVHQ

Supplementary Table 3. Melting temperature summary. Thermostability profile of humanized anti-pTag and mutant variants determined by nanotemp-DLS thermal ramping study.

Humanized anti-pTag scFv mutant variants melting temperatures

Humanized anti-pTag scFv mutant	Tm [°C]
NG (WT)	63.4
DG	62.4
GG	62.6
QG	62.2
SG	64.3
NA	60.8

Supplementary Table 4. Lead drug candidate BiA9*2_HF Off-target screening top hits. Summary of top target hits in large panel off-target screening for BiA9*2_HF. HEK cells were transfected to expressing the top target hits on the cell surface and ZsGreen only transfected cells functioning as negative control with fold changes in detection signal calculated against it. A Rituximab anti-CD20 biosimilar was used as positive control towards CD20, and assay buffer incubated with detection antibody as background control. Cut-off values based on fold changes for determination of binding strength described in legend below. Cut-off values based on fold changes for determination of binding strength is based on fluorescence intensity (FI) relative ZsGreen transfected HEK cells only. A Fold change of I) 2-2.5 Is classified as low confidence signal with Very weak FI binding, II) 2.6-4.0 classified as weak FI binding, III) 4.1-10 classified as medium FI binding and IV) >10 as strong FI binding. NT = Not tested

	**Median Fluorecent Intensity (MFI) AF647 anti-hIgGFc (Fold change from ZsGreen-only transfected HEK293 cells)**											
	**CD40 Isofrom 1**				**CD40 Isofrom 2 (secreted)**				**CD40 Isofrom 2 (Tethered secreted)**			
	**Replicate 1**		**Replicate 2**		**Replicate 1**		**Replicate 2**		**Replicate 1**		**Replicate 2**	
	**Median MFI**	**Fold**	**Median MFI**	**Fold**	**Median MFI**	**Fold**	**Median MFI**	**Fold**	**Median MFI**	**Fold**	**Median MFI**	**Fold**
**BiA9*2_HF (5 µg/mL) +AF647 anti-hIgG Fc**	**691645**	**555.9**	**649130**	**521.7**	1164	0.9	1237	1	**6065**	**4.9**	**6490**	**5.2**
**Assay Buffer +AF647 anti-hIgG Fc**	1133	1	1132	1	1186	1	1290	1.1	1208	1	1151	1
**Rituximab anti-CD20 biosimilar (1µg/mL) + AF647 anti-hIgG Fc**	1195	1	NT	NT	1214	1	NT	NT	1190	1	NT	NT

	**Median Fluorecent Intensity (MFI) AF647 anti-hIgGFc (Fold change from ZsGreen-only transfected HEK293 cells)**											
	**IGHM**				**CD20**				**HEKS (ZsGreen Only)**			
	**Replicate 1**		**Replicate 2**		**Replicate 1**				**Replicate 1**		**Replicate 2**	
	**Median MFI**	**Fold**	**Median MFI**	**Fold**	**Median MFI**		**Fold**		**Median MFI**	**Fold**	**Median MFI**	**Fold**
**BiA9*2_HF (5 µg/mL) +AF647 anti-hIgG Fc**	1233	1	1238	1	1306		1		1233	1	1256	1
**Assay Buffer +AF647 anti-hIgG Fc**	1125	0.9	1200	1	1142		1		1157	1	1218	1
**Rituximab anti-CD20 biosimilar (1µg/mL) + AF647 anti-hIgG Fc**	1127	1	NT	NT	**370950**		**314**		1183	1	1183	1
