# Supplementary material for: A bispecific CD40 agonistic antibody allowing for antibody-peptide conjugate formation to enable cancer-specific peptide delivery, resulting in improved T Cell proliferation and anti-tumor immunity in mice
Source: Nat Commun. 2024 Nov 5;15:9542. doi: 10.1038/s41467-024-53839-5 (PMC11538452; doi:10.1038/s41467-024-53839-5)
Supplement: Supplementary file 3 — Description of additional supplementary files [file 41467_2024_53839_MOESM3_ESM.pdf]

Description of Additional Supplementary Files

Supplementary Data file 1

Description: Amino acid sequences of Monospecific and Bispecific variants generated. Sequence name related to Table 1 followed by heavy chain (HC) and light chain (LC) sequences listed for respective construct.

Supplementary Data file 2

Description: Oligonucleotide sequences of Monospecific and Bispecific variants generated. Sequence name related to Table 1 followed by heavy chain (HC) and light chain (LC) sequences listed for respective construct.
